# Relationship of the Pine Growth Promoting *Pantoea eucalypti* FBS135 with Type Strains *P. eucalypti* LMG 24197^T^ and *P. vagans* 24199^T^

**DOI:** 10.3390/life11070608

**Published:** 2021-06-24

**Authors:** Chunyue Wei, Zhongwen Song, Yiming Lu, Yinjuan Zhao, Ben Fan

**Affiliations:** College of Forestry, Nanjing Forestry University, Nanjing 210037, China; chunyuewei@njfu.edu.cn (C.W.); zwsong@njfu.edu.cn (Z.S.); luym@gempharmatech.com (Y.L.); zhaoyinjuan@njfu.edu.cn (Y.Z.)

**Keywords:** endophyte, *Pantoea eucalypti* FBS135, phylogenetic analysis, nitrogen fixation, stable isotope, collinearity analysis

## Abstract

Endophytes in woody plants are much less understood. *Pantoea* strain FBS135 is an endophytic bacterium isolated from *Pinus massoniana* with the ability to promote pine growth significantly. In this study, we demonstrated that FBS135 has the astonishing ability of low nitrogen tolerance but no ability of nitrogen fixation. To exactly determine the phylogenetic status of FBS135, we sequenced the whole genomes of *P. eucalypti* LMG 24197^T^ and *P. vagans* 24199^T^, type strains of two *Pantoea* species, which are evolutionarily closest to FBS135. *P. eucalypti* LMG 24197^T^ contained a single chromosome of 4,035,995 bp (C+G, 54.6%) plus three circular plasmids while LMG 24199^T^ comprises a single circular chromosome of 4,050,173 bp (C+G, 55.6%) and two circular plasmids. With the genomic information, FBS135 was finally identified as a *P. eucalypti* strain, although it showed some different physiological traits from the two type strains. Comparative genomic analyses were performed for the three strains, revealing their common molecular basis associated with plant lifecycle as well as the differences in their gene arrangements relating to nitrogen utilization.

## 1. Introduction

Having been studied for decades, plant endophytes generally refer to those microorganisms that can colonize intercellular spaces or cells of plants and establish a symbiotic relationship with their host plants [1]. In recent years, Hallmann et al. raised a more practical definition of endophyte: Microorganisms separated from plant tissues that have been subjected to strict surface sterilization can be regarded as endophytes so long as they do not cause disease to the host plants [2,3]. Endophytes are widespread in plants. It is assumed that “plants without plant endophytes are a rare exception” [4].

Endophytes can promote plant growth through direct or indirect actions [5,6,7,8,9]. For example, endophytes can directly promote plant growth by producing and regulating various plant hormones such as auxin, cytokinin, or ethylene. Some endophytes can synthesize 1-aminocyclopropane-1-carboxylate (ACC) deaminase, which cleave to ACC in higher plants, thus reducing endogenous ethylene level. In addition, a large group of endophytes can obtain nitrogen, phosphorus, or iron from the environment and share with their hosts. Indirect beneficial mechanisms of endophytes not only include the production of antibiotics or volatile compounds, which suppress phytopathogens, but also the induction of systemic resistance to biotic pathogens and systemic tolerance to abiotic stress. Due to the various benefits they provide to host plants, endophytes have been drawing more and more attention in recent years.

*Pantoea* is a class of Gram-negative bacteria, which is ubiquitous in the environment. Many *Pantoea* strains are common endophytes [10,11]. The benefits of *Pantoea* to plants are similar to other endophytes as stated above. Intriguingly, some endophytic *Pantoea* strains isolated from sugarcane, sweet potato, rice, or other plants seem to share the trait of biological nitrogen-fixing activity [12,13,14,15]. For instance, *P. agglomerans* YS19, which has the ability to significantly promote the growth of rice, was verified for its nitrogen fixation function by acetylene reduction assay and ^15^N isotope labeling experiments. Thus, it is regarded suitable to be developed as a biological regulation agent [14]. Another endophyte *Pantoea sp.* 9C, isolated from sugarcane, can not only increases amino acid contents within sugarcane shoot tissues [16], but also significantly enhance the fresh weight of seedlings, the height of shoots, and both the length and number of rice seedlings’ roots [15]. This strain also has biological nitrogen fixation ability [13].

To date, research on endophytes mainly focuses on agricultural plants; only a few reports were performed on wood plants. *Pantoea* strain FBS135 is an endophytic bacterium isolated from the shoot of a healthy *Pinus massoniana* in Nanjing, China [17]. The greenhouse experiments evidenced that FBS135 can not only promote the growth of seedlings of *P. massoniana*, but also enhance the tolerance of pine seedlings under stress. Our earlier phylogenetic analysis revealed that FBS135 should be a member of *P. eucalypti*, a species firstly identified from eucalyptus in Uruguay [18]; however, the analysis was conducted solely based on its 16S rRNA sequences. Given the complexity of *Pantoea* genus [19] together with some different characters between FBS135 and *P. eucalypti*, we cannot exclude the possibility that FBS135 may be a novel species.

Nowadays, whole-genome information has become a requirement for exact taxonomic identification of prokaryotes. To clarify the taxonomic status of FBS135, in this study, we examined some important biological features of FBS135 and sequenced the whole genomes of the type strains for two species, which are pretty close to FBS135. Comparative genomic analysis was performed to reveal their genetic relationship.

## 2. Materials and Methods

### 2.1. Bacterial Strains and Media

The *Pantoea* strains and *Azotobacter chroococcum* ACCC10218 were routinely grown in LB medium unless otherwise stated. Ashby nitrogen-free medium (1 L): 10 g Mannitol, 0.2 g KH_2_PO_4_, 0.2 g MgSO_4_·7H_2_O, 0.2 g NaCl, 0.1 g CaSO_4_·2H_2_O, 5 g CaCO_3_, and 15 g Agar. NFDM nitrogen-free medium (1 L): 20 g sucrose, 0.015 g FeSO_4_·7H_2_O, 0.5 g MgSO_4_, 3.4 g KH_2_PO_4_, 0.01 g NaCl, 0.005 g Na_2_MoO_4_·2H_2_O, and 12.06 g K_2_HPO_4_ [20]. Gaseous Nitrogen-^15^N_2_(Enrichment > 99 atom%) was purchased from Shanghai Engineering Research Center of Stable Isotope.

### 2.2. Biological Assay

Physiological and biochemical tests were performed using GEN III MicroPlate following the instructions of the producer. FBS135 was grown on agar medium and then suspended in a special “gelling” inoculating fluid (IF) at the cell density of 90–98% T. Then the cell suspension was inoculated into the GEN III MicroPlate, 100 µL per well, and the MicroPlate was incubated at 30 °C to allow the phenotypic fingerprint to form [21,22]. The results were observed in 3–6 d.

### 2.3. Stable Isotope Labeling

Single colonies of FBS135 and *A. chroococcum* ACCC10218 were individually transferred to liquid medium and cultured at 30 °C and 200 rpm/min for four days. Then 50 μL of each bacterial culture were transferred to the plates of nitrogen-free media and spread evenly. The air containing ^15^N gaseous nitrogen (^15^N_2_) or normal air with ^14^N_2_ was used to incubate the plates. Three plates were placed in a sealed tank filled with the air containing 10% (*v/v*) ^15^N_2_ washed by sulfuric acid. Another three plates were placed in a tank filled with normal air. After both tanks had been incubated at 30 °C for 4 d, bacterial lawns were scraped off the plates and transferred into 50-mL centrifuge tubes. After being freeze-dried by an FD5-series freeze dryer (GOLD-SIM), the sample particle was passed through a clean 100-mesh sieve to obtain uniform bacteria powder, then packed in silver-foil cups and combusted within the reactor of an Organic Elemental Analyzer (FLASH 2000EA, Thermo Fisher Scientific, Bremen, Germany) using its existing reaction scheme for nitrogen analysis. Delta (15)N is determined using an isotope ratio mass spectrometer (DELTA V ADVANTAGE, Thermo Fisher Scientific, Bremen, Germany) [23].

### 2.4. Genome Sequencing

For DNA preparation, the bacteria were grown in LB at 37 °C with vigorous shaking. Cell pellets were collected and subjected to extraction with a Trizol^®^ agent (Thermo Fisher Scientific, Burlington, NJ, USA). Electrophoresis showed that the DNA size of both genomes was greater than 10,000 bp without smear. The value of OD 260/OD280 ranged from 1.87 to 1.89. Careful examination indicated that the DNAs prepared were of good quality for sequencing. Sequencing was performed with Illumina Hiseq plus PacBio RS II GridION or PromethION sequencers at Nextomics Biosciences Co., Ltd. (Wuhan, China). After read filtering and adapter trimming, the reads sequenced by Illumina were de novo assembled using SOAP de novo (v2.04) (SciCrunch Registry, RRID:SCR_005400) before being corrected by PacBio sequencing result. All corrected reads were further assembled with Celera Assembler 8.0. Scaffolds were verified with Illumina data, and gaps were closed with GapCloser v1.12.

Sequences of the genomes were blasted (blastn, parameter setting: evalue = 0.00001, outfmt = 6, max_target_seqs = 100,000) against the plasmid proprietary database. When the length of total alignments accounted for > 1/5 of the total sequence and the sequence was less than 1 Mb, it was judged as a plasmid sequence.

### 2.5. Annotation

Annotations of protein coding, tRNA, and rRNA genes were conducted with the NCBI Prokaryotic Genome Annotation Pipeline [24]. The rpsBLAST (parameter: -evalue 0.01 -seg no -outfmt 5, ver. 89) was carried out for COG annotation [25,26]; GO annotations (ver. 20180828) were performed using Blast2go; functional annotations of encoding proteins were completed with three databases, Refseq (ver. 89), Pfam (ver. 31.0), and TIGRFAMs (ver. 15.0); protein data were retrieved from TIGRFAMs, Pfam, and GO databases, and treated by Interproscan (parameters: -appl Pfam, TIGRFAM, SMART -iprlookup -goterms -tp -f TSV, ver. 5.30–69.0); encoding proteins searched from KEGG (ver. 87.0-r20180701) and Ref-seq databases went through Blastp (parameters: -evalue 1 × 10^−5^- outfmt 6 std qlen slen stitle -max_target_seqs 5) and BlastX.

### 2.6. Phylogenetic Analysis

The *rpoB, gyrB, infB,* and *atpD* gene sequences of all strains we used were downloaded from GenBank for MLSA [27]. The maximum likelihood method and the proximity method were employed for analysis. After the four genes were aligned with Clustal W, phylogenetic profiling was conducted using MEGA software (Version 6.0).

Average nucleotide identity (ANI) and average amino acid identity (AAI) matrices were calculated by the EDGAR software framework. Blast hits between orthologous genes of the core of the genomes were analyzed for their mean/median percent identity values. The species cut-off was set to be 95% for the ANI and AAI indices [28]. JSpecies WS online (http://jspecies.ribohost.com/jspeciesws/, accessed on 19 February 2020) was used to calculate ANIb (ANI based on BLAST+) and ANIm (ANI based on MUMmer) values by pairwise genome comparisons. The tetra-nucleotide signatures (TETRA) correlation indexes were also determined using the JSpeciesWS software (version 3.7.11) [29].

The genome-to-genome distance calculator (v2.1) (http://ggdc.dsmz.de/ggdc.php#, accessed on 6 May 2021) was employed for the genome-based DNA-DNA hybridization analysis. Distances were calculated by alignment tools BLAST+ (recommended) and MUMMER. Three calculative formulas were used: Formula 1 (HSP length/total length), formula 2 (identities/HSP length), and formula 3 (identities/total length).

The phylogeny distance analysis was completed with Composition Vector Tree (CVTree, v3) (https://www.github.com/ghzuo/cvtree, accessed on 6 May 2021). The genome sequences input were in (.faa) format so as to exploit basic parameters. Built-in genomes were chosen for comparison, while eight genomes from the NCBI database were selected as outgroups. The output results were in (.nwk) format and visualization was done using the software FigTree.exe (ver. 1.4.4) and iTOL (ver. 5) [30].

### 2.7. Collinearity Analysis

Genome-wide collinearity analysis was performed by the online tool MAUVE (Multiple Alignment of Conserved Genomic Sequence, v20150226). Results were saved in (.xmfa) format for subsequent analysis [31]. The text editor Sublime Text3 was used to view the backbone files containing both start and end information of the common and differential conservative region sequences. The generated topological guide_tree files in (.newick) format were read by FigTree.

## 3. Results

### 3.1. FBS135 Has Prominent Low Nitrogen Tolerance and Different Biochemical Characters from the Type Strains of P. vagans and P. eucalypti

**Figure 1:**
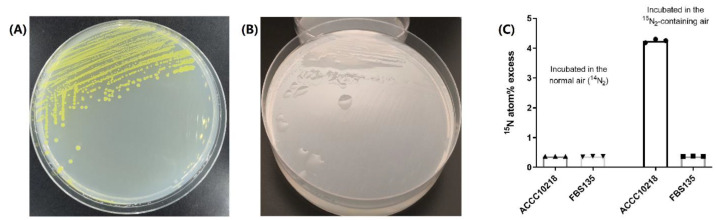
Low nitrogen tolerance ability of FBS135. (**A**) Morphology of FBS135 on Luria-Bertani plate. (**B**) Morphology of FBS135 on Ashby medium. (**C**) Detection of ^15^N content in bacterial cells of *P. eucalypti* FBS135 and *A. chroococcum* ACCC10218 incubated under different conditions. The two strains were cultured with normal air or the air containing ^15^N labeled N_2_.

Unlike the morphology on nutrient-rich LB plates (Figure 1A), FBS135 grew very well on nitrogen-free Ashby medium plates showing smooth and translucent colonies (Figure 1B), a typical character of slime layer. This phenomenon implies that FBS135 may have the ability of biological nitrogen fixation. However, we were unable to detect acetylene reduction activity with FBS135 (data not shown). A series of measurements were taken including growing FBS135 on a different medium recipe (NFDM medium) and substituting agar with agarose in plate preparation so as to avoid possible nitrogen pollution; nevertheless, FBS135 still grew vigorously after seven successive acts of streaking.

To directly determine whether FBS135 had N_2_ fixation activity, we performed a tracing test with ^15^N isotope, and using *A. chroococcus*, a well-known nitrogen fixer, as the positive control. The results showed that the ^15^N/^14^N ratio detected for *A. chroococcus* incubated in the air with ^15^N_2_ was around 10 times higher than the same strain incubated in the normal air with ^14^N_2_. By contrast, the ^15^N/^14^N ratio detected for FBS135 incubated in the ^15^N air showed no significant difference with that grown in the ^14^N normal air, both of which were below 0.5% and approximately equal to the level for *A. chroococcus* incubated in the normal air. These results evidenced that FBS135 was unable to fix gaseous nitrogen.

In our previous work, we identified FBS135 as a *P. eucalypti* strain based on its 16S RNA sequence. In the meantime, a taxonomic classification was performed using the Biolog^®^ system to learn more about its phenotypic patterns, including 71 carbon source utilization assays and 23 chemical sensitivity assays (Supplementary Materials Figure S1). The Biolog^®^ identification result showed that biochemical and physiological characteristics of FBS135 was closest to *P. vagans* followed by *P. eucalypti* (Figure S1). This is somewhat different from the taxonomic result according to 16S RNAs, although it was reasonable given the close relationship between *P. vagans* and *P. eucalypti*.

FBS135, *P. vagans* LMG 24199^T^, and *P. eucalypti* LMG 24197^T^ presented different capability of utilization of six carbon sources including Tween 40, α-D-lactose, gentiobiose, L-histidine, L-pyroglutamic acid, and also glucose (Table 1).

To further confirm this, an experiment on the carbon utilization of FBS135 was performed independently with glucose as the positive control (Figure S2F). On the sixth day, the optical density of FBS135 in gentiobiose culture reached its peak of approximately 5.0 (O.D.600 ≈ 5.0) (Figure S2C), followed by the culture fluid using Tween 40 or α-D-Lactose as the single carbon source (O.D._600_ ≈ 1.0) (Figure S2A,B). On the contrary, FBS135 failed entirely to grow when L-pyroglutamic acid or histidine was used as the single carbon source (Figure S2D,E). Taken together, the individual assays confirmed the difference between FBS135 and the two type strains concerning their availability in the presence of the five carbon substrates. Although the differences may be only strain-specific, it is important not to exclude the possibility that FBS135 may be a novel species, since it is well known that distinguishing the species within *Pantoea* only by 16S sequences is challenging [19].

### 3.2. WGS Analysis of the Two Type Strains and FBS135

**Figure 2:**
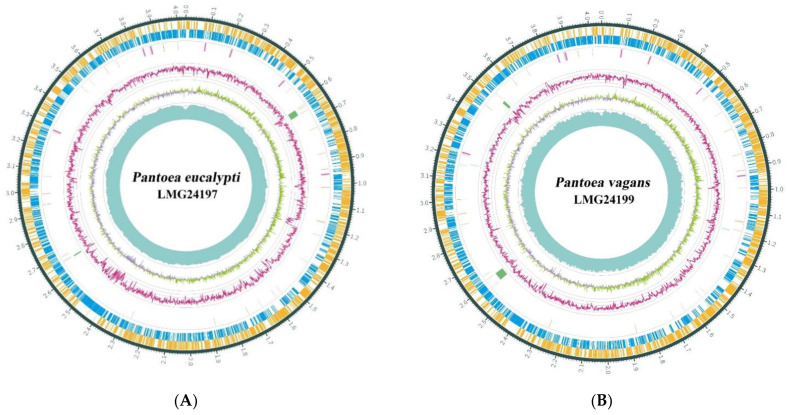
Genome structure diagram for *Pantoea eucalypti* LMG 24197^T^ and *Pantoea vagans* LMG 24199^T^. The circle diagrams from the outermost layer to the innermost layer show their sense strands, the negative sense strands, tRNAs, rRNAs, CRISPR-related genes, gene islands, GC content percentage, GC-skew, and genome sequencing depth. (**A**) Main genome structure diagram of *P. eucalypti* LMG 24197^T^. (**B**) Main genome structure diagram of *P. vagans* LMG 24199^T^ m.

To precisely pinpoint the taxonomic status of FBS135, we did a whole-genome sequencing of two type strains, *P. eucalypti* LMG 24197^T^ and *P. vagans* LMG 24199^T^, which are closely related to FBS135 according to 16S RNA profiling. The statistics showed that the qualities for both of the sequencing and the assembly were satisfactory (Table S1A,B). The genome of *P. eucalypti* LMG 24197^T^ comprises a single circular chromosome (4,035,995 bp, C+G, 54.6%) and three circular plasmids, named pVag1 (~529 kb), pVag2 (138 kb), and pVag3 (94 kb) (Figure 2A). The genome sequence and the three plasmids are deposited in the DDBJ/EMBL/GenBank database under the accession numbers NZ_CP045720, NZ_CP045721, NZ_CP045722, and NZ_CP04573, respectively. The genome of LMG 24199^T^ comprises a single circular chromosome (4,050,173 bp, C+G, 55.6%) and two circular plasmids, named pEuc1 (~560 kb) and pEuc2 (180 kb) (Figure 2B). The genome sequence and the three plasmids are deposited in the DDBJ/EMBL/GenBank database under the accession numbers NZ_CP038853, NZ_CP038854, and NZ_CP038855, respectively. Genome and plasmid information of the two strains have been summarized in Table 2. Different from the two strains, FBS135 has four plasmids, one (pPant4) of which seems to be responsible and indispensable for its growth [17].

Annotation analysis showed that the genome structures of *P. eucalypti* LMG 24197^T^ and *P. vagans* LMG 24199^T^ are generally similar yet with differences. *P. eucalypti* LMG 24197^T^ contained a total of 4424 complete CDSs, while *P. vagans* LMG 24199^T^ contained 4331 complete CDSs (Figure 2). *P. vagans* LMG 24199^T^ contained more 5S rRNA sequence (Table S2) and two CRISPR (Clustered Regularly Interspaced Short Palindromic Repeat Sequences)-related genes in a total size of 1705 bp (Table S3). No CRISPR-related genes were found in the genomes of LMG 24197^T^ or FBS135. In addition, *P. vagans* LMG 24199^T^ contains one more genomic island (Table 3).

The genomes of *P. eucalypti* LMG 24197^T^ and *P. vagans* LMG 24199^T^ were annotated against COG, GO, KEGG, Refseq, Pfam, and TIGRFAMs databases (Table S4). Furthermore, 3144 genes (71.07%) of *P. eucalypti* LMG 24197^T^ and 3180 genes (73.42%) of *P. vagans* LMG 24199^T^ were successfully annotated with COG (Figure S3). Both genomes comprise more than 100 genes of unknown function. Moreover, 2739 genes (61.91%) of *P. eucalypti* LMGq 4197T and 2776 (64.1%) genes of *P. vagans* LMG 24199^T^ were assigned to the GO database. The GO functional classification of the annotated proteins of the two genomes shared a high similarity. For both strains, the largest number of genes was assigned to the group of Biological Process, within which the sub-group of Metabolic Process (MP) and Cellular Process (CP) comprises the two highest numbers of genes (Figure S4). In addition, 2812 (63.56%) genes of *P. eucalypti* LMG 24197^T^ and 2842 (65.62%) genes of *P. vagans* LMG 24199^T^ were assigned to different KEGG pathways. For both strains, the pathway for membrane transports contained the largest number of their annotated genes (Figure S5). In general, the number of successfully annotated genes for *P. eucalypti* LMG 24197^T^ and *P. vagans* LMG 24199^T^ against different databases are similar.

Of note, in both genomes, we found neither *nifH*, the very conservative gene for nitrogenase, nor other common genes known to be involved in nitrogen fixation. Similarly, we did not find these genes in FBS135. These results confirm the inability of the three strains to fix inorganic nitrogen, which was just as consistent with the ^15^N-labelling experiment result above. Our results further showed that, similar to FBS135, both *P. eucalypti* LMG 24197^T^ and *P. vagans* LMG 24199^T^ could thrive on nitrogen-free Ashby plates after seven successive acts of streaking, indicating that the two strains also have strong ability of low nitrogen tolerance.

### 3.3. Phylogentic Analysis of the Two Type Strains and FBS135 Based on Genome Information

With the genome information above, we were able to make a more exact phylogenetic analysis for FBS135 together with *P. eucalypti* LMG 24197^T^ and *P. vagans* LMG 24199^T^. We firstly constructed the phylogenetic trees based on the conversional multilocus sequence analysis (MLSA) in order to compare the result with an earlier study where *P. eucalypti* LMG 24197^T^ and *P. vagans* LMG 24199^T^ were initially reported [18]. Four conserved genes (*rpoB, gyrB, infB,* and *atpD*) of 21 type *Pantoea* strains were used for the MLSA while *Budvicia aquatica* DSM 5075^T^ was used as the outgroup. According to the MLSA, FBS135, *P. eucalypti* LMG 24197^T^, and *P. eucalypti* LMG 24198 ^T^ were clustered into one branch (Table S5), which was basically consistent with the taxonomic result based on 16S rRNA sequences (Figure S6).

**Figure 3:**
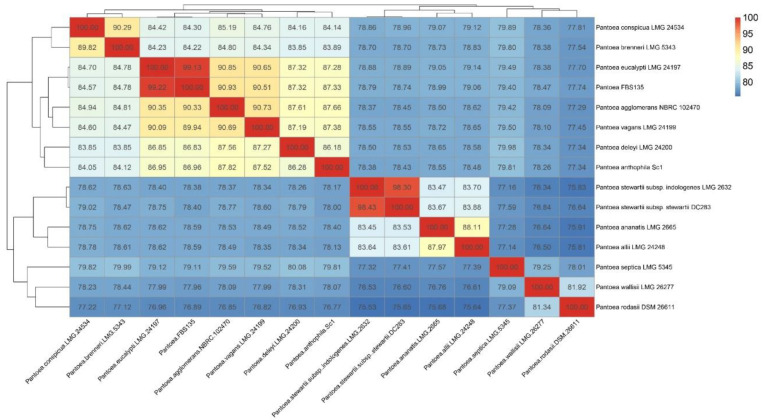
Heat map of ANI comparison between FBS135 and related strains.

ANI, TETRA, and dDDH are three popular methods that are used currently to discriminate a wide range of bacterial taxons. An ANIb value between *P. vagans* LMG 24199^T^ and *P. eucalypti* LMG 24197^T^ was 90.09%, confirming that they were different species. The ANIb value between FBS135 and *P. eucalypti* LMG 24197^T^ was greater than 99%, while the value between FBS135 with *P. vagans* LMG 24199^T^ was merely 89.94% (Figure 3). ANIm values between the strains were almost the same as the ANIb values. The TETRA value between FBS135 and *P. eucalypti* LMG 24197^T^ was 0.9997, greater than the corresponding cut-off value 0.998 (Table S6). With three different formulas, the digital DNA–DNA hybridization (dDDH) values of LMG 24197^T^ and LMG 24199^T^ were calculated to be 84.2%, 42.4%, and 75.9%, respectively. The dDDH between FBS135 and LMG 24199^T^ were 95.3%, 94.6%, and 96.9% respectively, all exceeding the threshold value 70%. On the contrary, the dDDH between FBS135 and LMG 24199^T^ were 69.6%, 42.3%, and 64.2%, respectively (Table S7), all less than 70%, indicating that FBS135 and LMG 24199^T^ were different species. With all clues above, it was concluded that FBS135 and *P. eucalypti* LMG 24197^T^ belonged to the same species.

It was interesting to know that from the perspective of GC mol% alone, the various *Pantoea* species showed obvious differences. The GC mol% differences between *P. eucalypti* LMG 24197^T^ and *P. vagans* LMG 24199^T^ is nearly 0.5%. The GC mol% differences between FBS135 and LMG 24197 is nearly 0.2%, while the difference between FBS135 and *P. vagans* LMG 24199^T^ is around 1% (Table S7). Therefore, the GC mol% differences again corroborated that FBS135 was phylogenetically closer to LMG 24197^T^ than to LMG 24199^T^.

Finally, we analyzed the taxonomic relationships of the strains with CVTree, a software that can construct phylogenetic trees based on whole-genome sequences but using a different algorithm from the above methods [32]. The result given by CVtree also showed that FBS135 and *P. eucalypti* LMG 24197^T^ were clustered into one clade (Figure S7), which is consistent with the results from the other methods above.

### 3.4. Comparative Analysis Reveals Genomic Differences between FBS135 and Its Relative Strains

**Figure 4:**
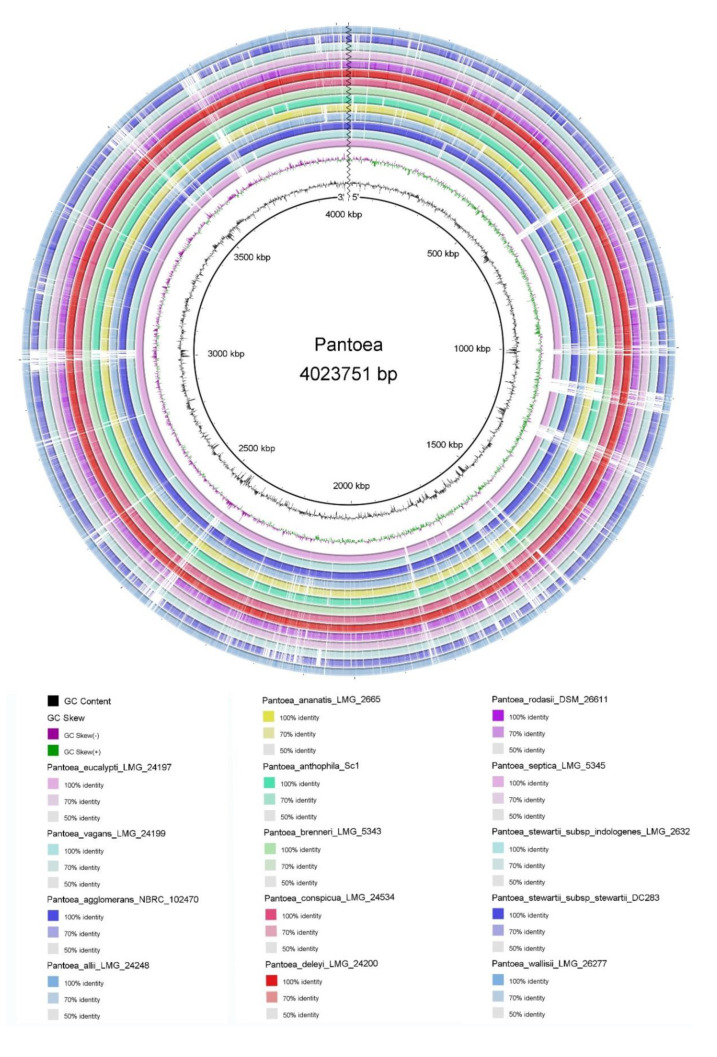
Genome-wide visualization results of FBS135 and some related strains.

To better understand genomic differences among FBS135 and its phylogenetic relatives, their genomes were visualized together (Figure 4). It is easy to see that the genome of FBS135 has a higher similarity to the genome of *P. eucalypti* LMG 24197^T^ than to the other *Pantoea* strains. Compared with FBS135, there are large fragment deletions at the region of 600 kb, 1000 kb, 1300 kb, and 3600 kb in these strains including *P. eucalypti* LMG 24197^T^ and *P. vagans* LMG 24199^T^. FBS135 shared no gene overlap with the two type strains at 65 regions of different size ranging from 20 bp to 40,776 bp. Five of these regions were larger than 10,000 bp with the longest one (40,776 bp, from 1,092,468 bp to 1,133,244 bp) containing a multitude of genes encoding MFS transporter, transcriptional regulator, phage regulatory protein, and a lot of hypothetical proteins of unknown functions.

Five proteins (VgrG, TssH, TssK, B9D02_RS17585, B9D02_RS17595) related to the type VI secretion system were detected in a large region of 23,860 bp (3,673,699 bp~3,697,559 bp). In another large region of 37,425 bp (1,241,124 bp~1,278,359 bp), dozens of phage-related genes were found, indicating their possible phage origin. It is noteworthy that two genes encoding host specificity protein J (B9D02_RS06210) and serine acetyltransferase (B9D02_RS06115) were found in this region. Both of the genes were identified to be involved in FBS135 ability to tolerate low nitrogen (data now shown). The serine acetyltransferase gene (B9D02_RS06115) was also present in *P. eucalypti* LMG 24197^T^ and *P. vagans* LMG 24199^T^, while the host-specific protein J gene (B9D02_RS06210) was only found in FBS135. Mechanisms of the two genes involved in low nitrogen tolerance remain unknown.

We aligned the genome sequences of the three strains according to their similarity and then split them into 1541 fragments of different sizes. FBS135 was found to have 65 exclusive fragments but lack 57 fragments present in the other two genomes; LMG 24197^T^ had 93 exclusive fragments but lacked 44 fragments present in the other two genomes; *P. vagans* LMG 24199^T^ had 368 exclusive fragments but lacked 445 fragments presented in the other two genomes. *P. vagans* LMG 24199^T^ contained much more DNA fragments than *P. eucalypti* LMG 24197T and FBS135.

**Figure 5:**
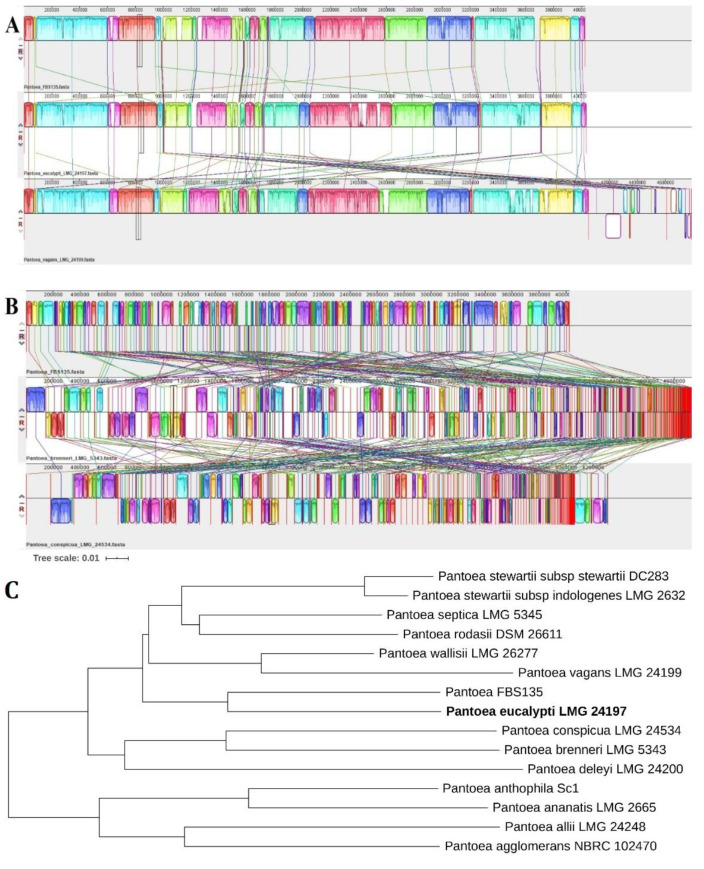
Comparative analysis revealed genomic difference between FBS135 and its relative strains. Collinearity analysis results of FBS135 with *P. eucalypti* LMG24197^T^ and *P. vagans* LMG24199^T^ (**A**) and FBS135 with *P. brenneri* LMG5343^T^ and *P. conspicua* LMG24534^T^ (**B**). Simulated topology tree for FBS135 and the related strains according to their collinear analysis (**C**).

The collinearity analysis demonstrated that the genome structures of FBS135 and *P. eucalypti* LMG 24197^T^ were almost identical. Their major fragments were well matched without obvious gene inversion or insertion (Figure 5A). The collinearity degree between FBS135 and *P. vagans* LMG 24199^T^ was also high, although some inverted or rearranged fragments were detected. The genome collinearity between the FBS135 and other strains, for example, strain *P. brenneri* LMG5343^T^ or *P. conspicua* LMG24534^T^, was poor. A large number of rearrangement events such as fragment inversion, deletion, translocation, or insertion were found in their genomes (Figure 5B). It is noteworthy that there was a large volume of differences between gene arrangements of *P. brenneri* LMG 5343^T^ and FBS135, although they were phylogenetically close with a 16S rDNA similarity as high as 99.2%.

According to these collinearity analysis results, a rootless phylogenetic topology tree was generated (Figure 5C), where FBS135 and *P. eucalypti* LMG 24197^T^ were clustered in the same clade while LMG 24199^T^ was in another clade. Other type strains were even distant from FBS135. Such results were consistent with their phylogenetic relationships as analyzed above.

## 4. Discussion

In this study, the complete genomes of *P. eucalypti* LMG 24197^T^ and *P. vagans* LMG 24199^T^ have been sequenced and analyzed. With the genome information, we precisely determined the taxonomic status of *Pantoea sp.* FBS135, an endophyte isolated from *Pinus massoniana* with evidenced ability of promoting pine growth and stress tolerance [17]. We also demonstrated that FBS135 can tolerate an extremely low nitrogen environment without fixing nitrogen in the air. Comparative genomic analysis was performed for the two type strains and FBS135 revealed their close relationships and differences.

Compared with a pyramid of studies on symbiotic relationship between endophytic bacteria and agricultural plants, interactions between endophyte and forest plants are scarcely investigated. The significance of endophytes on forest plants may thus be underestimated, especially considering the huge influence of forest system on global ecology [33,34,35]. Studies on endophytes are also important for the production of economic trees. For example, endophytes have been demonstrated to have a remarkable performance as biocontrol agents and as plant growth promoters for grapevines [36,37]. In another example, *P. agglomerans* SWg2, an endophytic bacterium isolated from healthy mulberry roots, has been proven to not only promote mulberry growth and vitality, but also protect mulberry trees from bacterial pathogens [38].

We have previously reported the beneficial effect of FBS135 on pine growth [17]; however, the phylogenetic status of FBS135 was only roughly determined on the basis of 16S sequence, which will hinder further studies on it. In recent years, precise identification of a bacterium using whole-genome sequence has been proposed to be the standard [28,39]. Unfortunately, this has not been applied to FBS135 due to the absence of whole-genome information of type strains that are phylogenetically close to FBS135. It is challenging to distinguish species within *Pantoea* genera merely by 16S rRNA sequences. For instance, James T. Tambong found that many *Pantoea* strains have been previously misidentified by using MLSA. [40] The evolutionary relationship between *P. eucalypti* and *P. vagans* are also very close. In the GenBank, there are multiple genome sequences identified as *P. vagans*, which has been corrected to be *P. eucalypti* after a complete phylogenetic analysis. Therefore, it is extremely necessary to sequence the whole genomes of LMG 24197^T^ and LMG 24199^T^, which do not only provide valuable information for the two type stains themselves, but also help to solve the taxonomic status of FBS135.

Widely distributed in the world and as one of the main tree species for artificial forestation in China, pine trees play an important role in the world’s forest system. Pine can tolerate barren soil and are often cultivated in barren soil. Ecological studies have shown that the nitrogen in the region is not enough to support the biomass of pine trees [41]. Thus, the nitrogen shortage has been a mystery for a long time. In recent years, some researchers have put forward convincing evidence that nitrogen-fixing endophytes in pine trees may be a main contributor responsible for the “mysterious” nitrogen source [33,34,42,43,44], although a deep understanding of the endophytes is still lacking. Mechanisms of biological nitrogen fixation in a highly oxygen-producing environment like pine needles also remains unclear. The possibility should not be excluded that some endophytes, during the long process of life evolution, may have formed special nitrogenases, which are not sensitive to oxygen.

As a class of typical endophytic bacteria, some *Pantoea* strains are found to have nitrogen fixation ability [12,13,14,15,45]. As the main objective of our study, *Pantoea sp.* FBS135 can grow vigorously on plates of two different nitrogen-free media (Ashby and NFDM), even after seven acts of successive streaking. Both mediums are prepared with analytically pure chemicals and the main carbon sources are different. Furthermore, we used agarose instead of agar to prepare plates in order to reduce trace nitrogen impurities. In either case, FBS135 grew very well. The odds are slim that FBS135 grew with nitrogen provided by the trace impurities from somewhere, which, therefore, caused us to speculate whether FBS135 has the ability of biological nitrogen fixation. However, the acetylene reduction assays with FBS135 showed a negative result. In addition, we failed to detect the presence of *nifH*, the most conserved gene for nitrogenase, in the genome of FBS135. Both facts appeared in contradiction to the speculation, indicating that either the speculation is incorrect, or FBS135 has a different nitrogen fixation mechanism from the traditional concepts.

In order not to miss a possible important discovery like novel nitrogenase, we exploited an additional method with stable isotope ^15^N labeling to determine whether FBS135 has the ability of N_2_ fixation. The detection with ^15^N isotope is extremely sensitive and, more importantly, it is a direct measurement, in contrast to the acetylene reduction assay, which may lead to a negative result in the case of a novel nitrogenase that cannot reduce acetylene but only N_2_. Unfortunately, the results proved that FBS135 cannot fix gaseous nitrogen. Its growth in/on strictly formulated nitrogen-free medium could be supported by extremely trace impurities in chemicals or agar/agarose.

## 5. Conclusions

In summary, in this study, we report the complete genome sequence of *P. eucalypti* LMG 24197^T^ and *P. vagans* 24199^T^. As two type strains, their genome information is valuable for taxonomic identification of *Pantoea* strains, which are widely distributed in the environment but not identified at a species level [19,40]. In addition, the pine endophyte FBS135 was precisely identified with the genome information of the two strains. We also demonstrated that FBS135 failed to fix nitrogen but has a strong ability of tolerating extremely low nitrogen nutrients. In the future, more in-depth studies will be focused on the ability of FBS135′s tolerance to low nitrogen, which can help to elucidate the adaptability of this type of bacteria to the environment and shed light on the mechanisms of their symbiosis with host plants.

## Figures and Tables

**Table 1:** Carbon utilization difference between FBS135 and two related strains.

Characteristic	*P. vagans* LMG 24199^T^	*P. eucalypti* LMG 24197^T^	FBS135
Tween 40	+	+	(+)
α-D-Lactose	-	+	(+)
Gentiobiose	(+)	-	+
L-Histidine	+	+	-
L-pyroglutamic acid	-	+	-

Note: +, 90–100% of strains positive in 3–6 days; (+), weakly positive; -, 90–100% of strains negative.

**Table 2:** Genomic and plasmid information of *P. eucalypti* LMG 24197^T^ and *P. vagans* LMG 24199^T^ and FBS135.

Strain	Type	Length (bp)	GC Content (%)	Topology
*Pantoea vagans* LMG24199^T^	genome	4,050,173	55.6	circular
	pVag1	559,692	54.0	circular
	pVag2	180,464	53.1	circular
*Pantoea eucalypti* LMG 24197^T^	genome	4,035,995	54.6	circular
	pEuc1	529,303	52.3	circular
	pEuc2	138,725	50.9	circular
	pEuc3	94,967	52.2	circular
*P. eucalypti* FBS135	genome	4,023,751	56.2	circular
	pPant1	535,553	52.4	circular
	pPant2	199,372	50.7	circular
	pPant3	133,127	51.7	circular
	pPant4	73,562	53.3	circular

**Table 3:** Gene structure statistics of *P. eucalypti* LMG 24197^T^ and *P. vagans* LMG 24199^T^.

Strain	*P. vagans* LMG 24199^T^			*P. eucalypti* LMG24197^T^		
Type	Number	Length (bp)	% Genome	Number	Length (bp)	% Genome
tRNA	80	6268	0.13	80	6292	0.13
16S rRNA	7	10,700	0.22	7	10,702	0.22
23S rRNA	7	20,337	0.42	7	20,355	0.42
5S rRNA	8	920	0.02	7	805	0.02
CDS	4331	4,147,020	86.57	4424	4,127,715	86.01
CRISPR	2	1705	0.04	0	0	0
genomic_ island	2	50,847	1.06	3	34,373	0.72

## Data Availability

Not applicable.

## Supplementary Materials

The following are available online at https://www.mdpi.com/article/10.3390/life11070608/s1, Figure S1: ‘Phenotypic Fingerprint’ of FBS135 after six-day cultivation, Figure S2: Utilization of single carbon source by FBS135 in six days, Figure S3: Classification and statistics of COG function of genome -encoded proteins, Figure S4: Classification of GO functionally encoded proteins, Figure S5: Statistics of KEGG functional classification of genome-encoded proteins, Figure S6: Phylogenetic tree constructed based on MLSA with four conserved genes, Figure S7: Phylogenetic analysis based on the whole genome sequence of FBS135; Table S1A: Sequencing data statistics, Table S1B: Assembly result statistics, Table S2: ncRNA prediction result statistics, Table S3: CRISPR prediction result, Table S4: Functional annotation statistics of genome-encoded proteins, Table S5: Strain information for phylogenetic analysis, Table S6: Key parameters of phylogenetic analysis between FBS135 and *P. eucalypti* LMG 24197^T^, Table S7. Digital DNA-DNA hybridization results of FBS135 and two type strains.
