# Supplementary material for: Relationship of the Pine Growth Promoting Pantoea eucalypti FBS135 with Type Strains P. eucalypti LMG 24197T and P. vagans 24199T
Source: Life (Basel). 2021 Jun 24;11(7):608. doi: 10.3390/life11070608 (PMC8307521; doi:10.3390/life11070608)
Supplement: Supplementary file 1 [file life-11-00608-s001.zip › supp.pdf]

Supplementary Tables

Table S1. A Sequencing data statistics.

**Strain**	**Pantoea Vagans LMG 24199T**		**Pantoea Eucalypti LMG 24197T**	
Type	Raw Reads	Filtered Reads	Raw Reads	Filtered Reads
Bases (bp)	1,432,460,598	1,378,041,586	2,519,894,578	2,517,577,992
Reads number	126,071	114,716	130,822	126,539
Reads mean length (bp)	11,362	12,012	19,262	19,895
Reads N50 (bp)	14,359	14,481	31,522	31,553
Longest Reads (bp)	250,802	137,167	202,879	202,879

Table S1. B Assembly result statistics.

**Strain Name**	**P. Eucalypti LMG 24197T**	**P. Vagans LMG 24199T**
Genome size (bp)	4,798,990	4,790,329
Contig Number	4	3
Contig N50 (bp)	4,035,995	4,050,173
Longest contig(bp)	4,035,995	4,050,173
Shortest contig(bp)	94,967	180,464

Table S2. ncRNA prediction result statistics.

**strain**	**Pantoea Vagans LMG 24199T**		**Pantoea Eucalypti LMG24197T**	
Type	Number	Length (bp)	Number	Length (bp)
tRNA	80	6,268	80	6,292
rRNA	22	31,957	21	31,862
other	48	7,387	46	7,293
regulatory	40	6,155	50	7,688

Table S3. CRISPR prediction result.

**Strain**	**Contig**	**ID**	**Start**	**End**
Pantoea vagans LMG 24199T	tig00001	CRISPR array 1	1197759	1198885
	tig00001	CRISPR array 2	3172624	3173201

Table S4. Functional annotation statistics of genome-encoded proteins.

**Database**	**FBS135**	**Pantoea Vagans LMG 24199T**	**Pantoea Eucalypti LMG 24197T**
COG	2972	3180	3144
KEGG	2356	2842	2812
GO		2776	2739
Refseq		4273	4357
Pfam		3912	3863
TIGRFAMs		1526	1492
all databases		1228	1215
at least one databases		4273	4359
Overall		4331	4424

Table S5. Strain information for phylogenetic analysis.

Rank	Name	Strain	Accession	16S rDNA Similarity (%)
1	Pantoea eucalypti	LMG 24197	EF688009	99.33
2	Pantoea vagans	LMG 24199	EF688012	99.56
3	Flavobacterium acidificum	LMG 8364	JX986959	99.35
4	Pantoea brenneri	LMG 5343	MIEI01000169	99.21
5	Pantoea ananatis	LMG 2665	JMJJ01000010	99.50
6	Pantoea anthophila	LMG 2558	EF688010	100
7	Pantoea conspicua	LMG 24534	MLFN01000105	99.35
8	Pantoea allii	LMG 24248	AY530795	99.28
9	Pantoea agglomerans	DSM 3493	AJ233423	99.14
10	Pantoea deleyi	LMG 24200	EF688011	99.35
11	Pantoea indologenes	LMG 2632	JPKO01000033	98.35
12	Pantoea stewartii	LMG 2715	Z96080	98.28
14	Pantoea rodasii	LMG 26273	MLFP01000054	97.35
18	Leclercia adecarboxylata	NBRC 102595	BCNP01000062	97.28
19	Pantoea septica	LMG 5345	MLJJ01000077	97.5
20	Pantoea coffeiphila	Ca04	KJ427829	98
21	Pantoea rwandensis	LMG 26275	MLFR01000061	96.85

Table S6. Key parameters of phylogenetic analysis between FBS135 and *P. eucalypti* LMG 24197^T.

Category	Calculated Value	Cut-off Value
MLSA (%)	99	97
gDDH (%)	94.6*	70
ANIb(%)	99.22	96
ANIm (%)	99.26	96
TETRA	0.9997	0.998

*: The lowest value calculated with the formula 2.

Table S7. Digital DNA-DNA hybridization results of FBS135 and two type strains.

	Formula	DDH	Model C.I.	Distance	Prob. DDH >= 70%	G+C Difference
LMG 24197T /LMG 24199T	1	84.2	[80.5 - 87.4%]	0.111	94.49	1.05
LMG 24197T /LMG 24199T	2	42.4	[39.9 - 44.9%]	0.0918	4.78	1.05
LMG 24197T /LMG 24199T	3	75.9	[72.5 - 79.1%]	0.1926	90.29	1.05
FBS135/LMG 24197T	1	95.3	[93.1 - 96.9%]	0.0492	98.73	0.29
FBS135/LMG 24197T	2	94.6	[92.9 - 95.9%]	0.0071	97.11	0.29
FBS135/LMG 24197T	3	96.9	[95.5 - 97.9%]	0.056	99.91	0.29
FBS135/LMG 24199T	1	69.6	[65.6 - 73.2%]	0.1859	73.18	0.48

FBS135/LMG 24199T	2	42.3	[39.8 - 44.9%]	0.092	4.71	0.48
FBS135/LMG 24199T	3	64.2	[60.9 - 67.4%]	0.2608	46.06	0.48
