# Supplementary material for: Relationship of the Pine Growth Promoting Pantoea eucalypti FBS135 with Type Strains P. eucalypti LMG 24197T and P. vagans 24199T
Source: Life (Basel). 2021 Jun 24;11(7):608. doi: 10.3390/life11070608 (PMC8307521; doi:10.3390/life11070608)
Supplement: Supplementary file 1 [file life-11-00608-s001.zip › Suppl. Figures.pdf]

Supplemental figures:

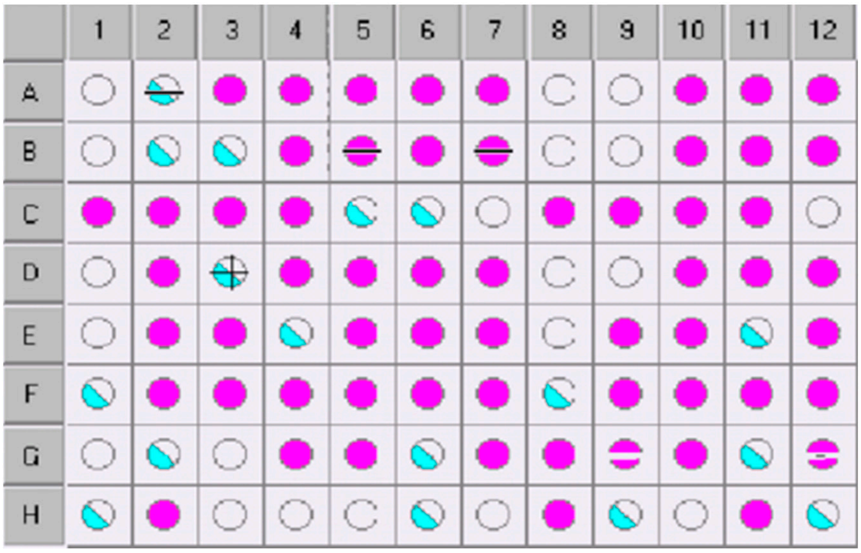

Figure S1. 'Phenotypic Fingerprint' of FBS135 after six-day cultivation. The Biolog GEN III MicroPlate analyzes FBS135 in 94 phenotypic tests: 71 carbon source utilization assays (columns 1-9) and 23 chemical sensitivity assays (columns10-12).

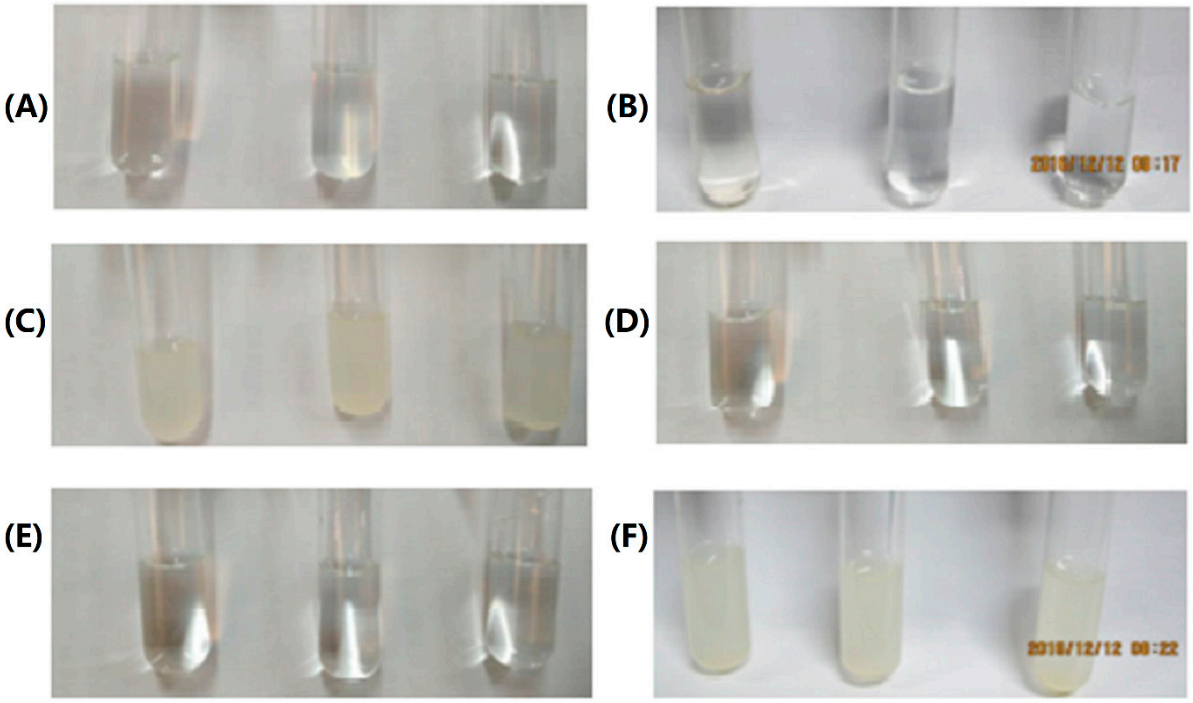

Figure S2. Utilization of single carbon source by FBS135 in six days. (A: Tween 40, B: α-D-Lactose, C: Gentiobiose, D: L-histidine; E: L-pyroglutamic acid; F: Glucose).

Accordingly, the original S1 have been renamed as S3; original S2 have been renamed as S4; original S3 have been renamed as S5.

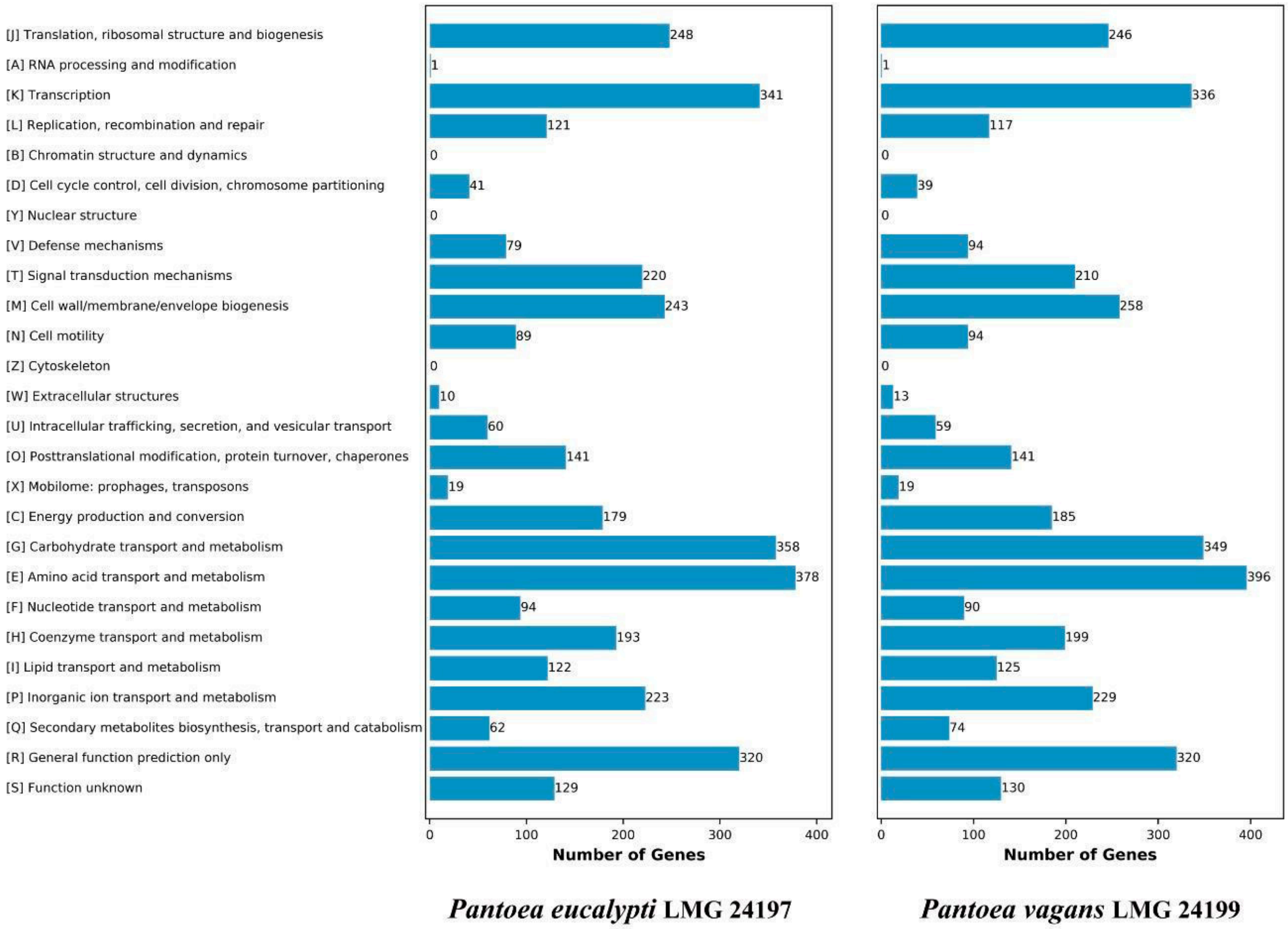

Figure S3. Classification and statistics of COG function of genome -encoded proteins.

Pantoea vagans LMG 24199

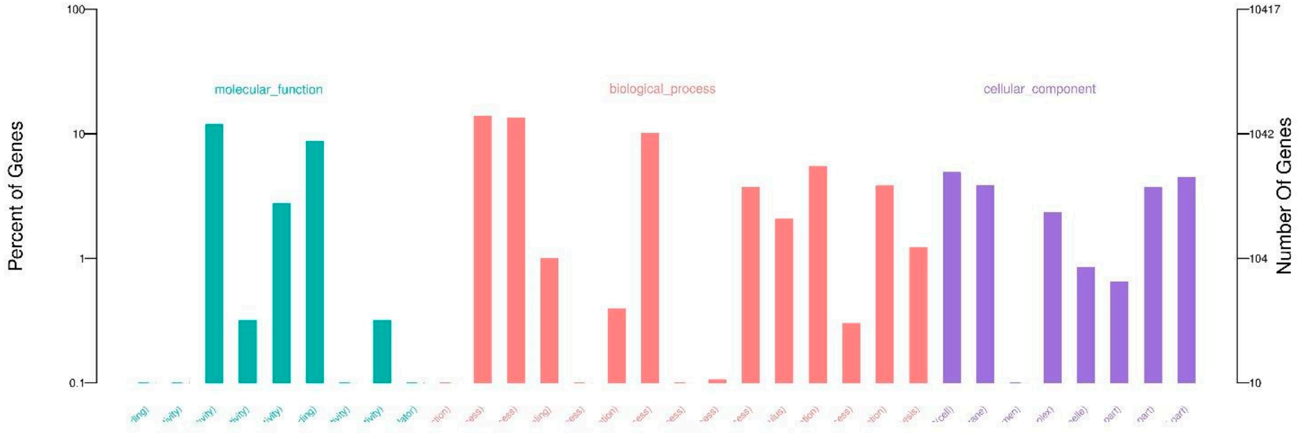

Pantoea eucalypti LMG 24197

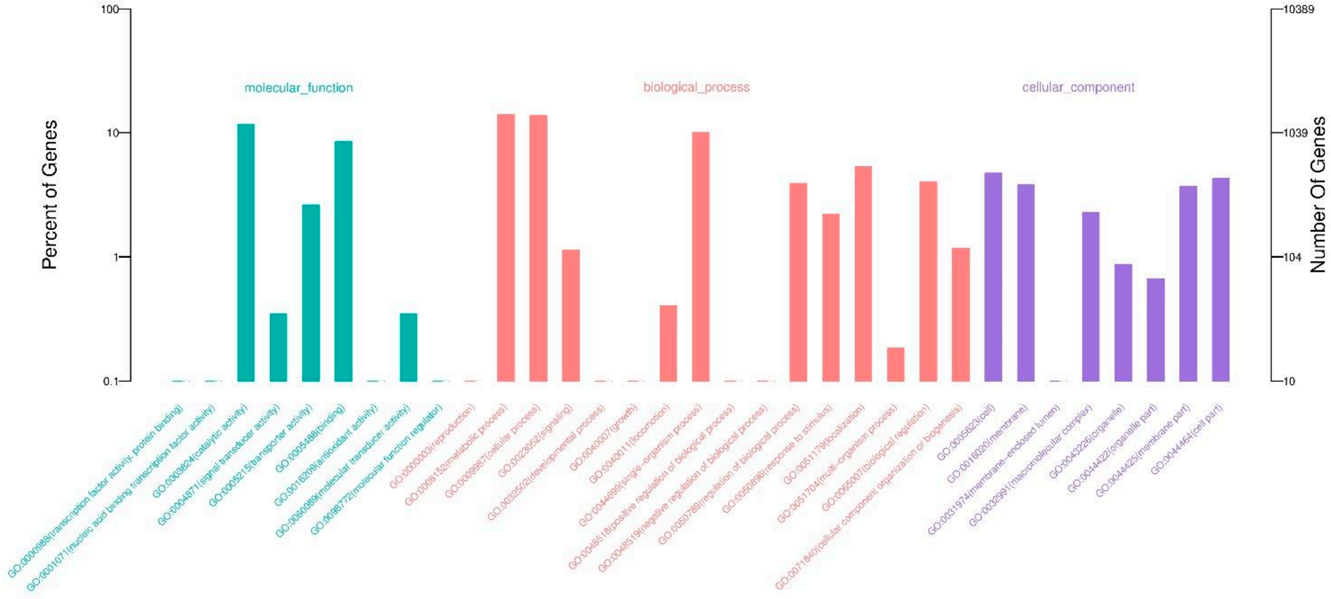

Figure S4. Classification of GO functionally encoded proteins.

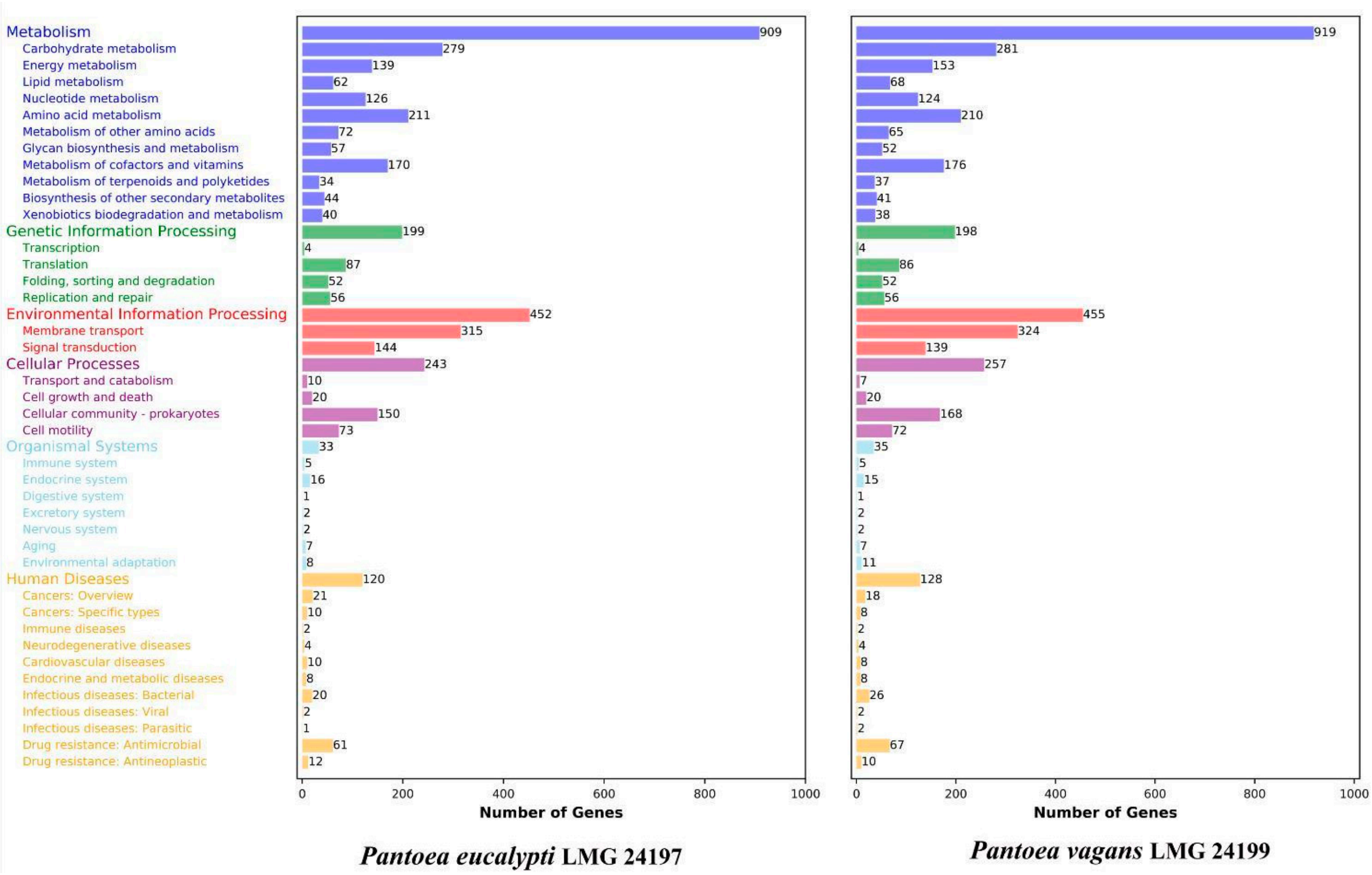

Figure S5. Statistics of KEGG functional classification of genome-encoded proteins.

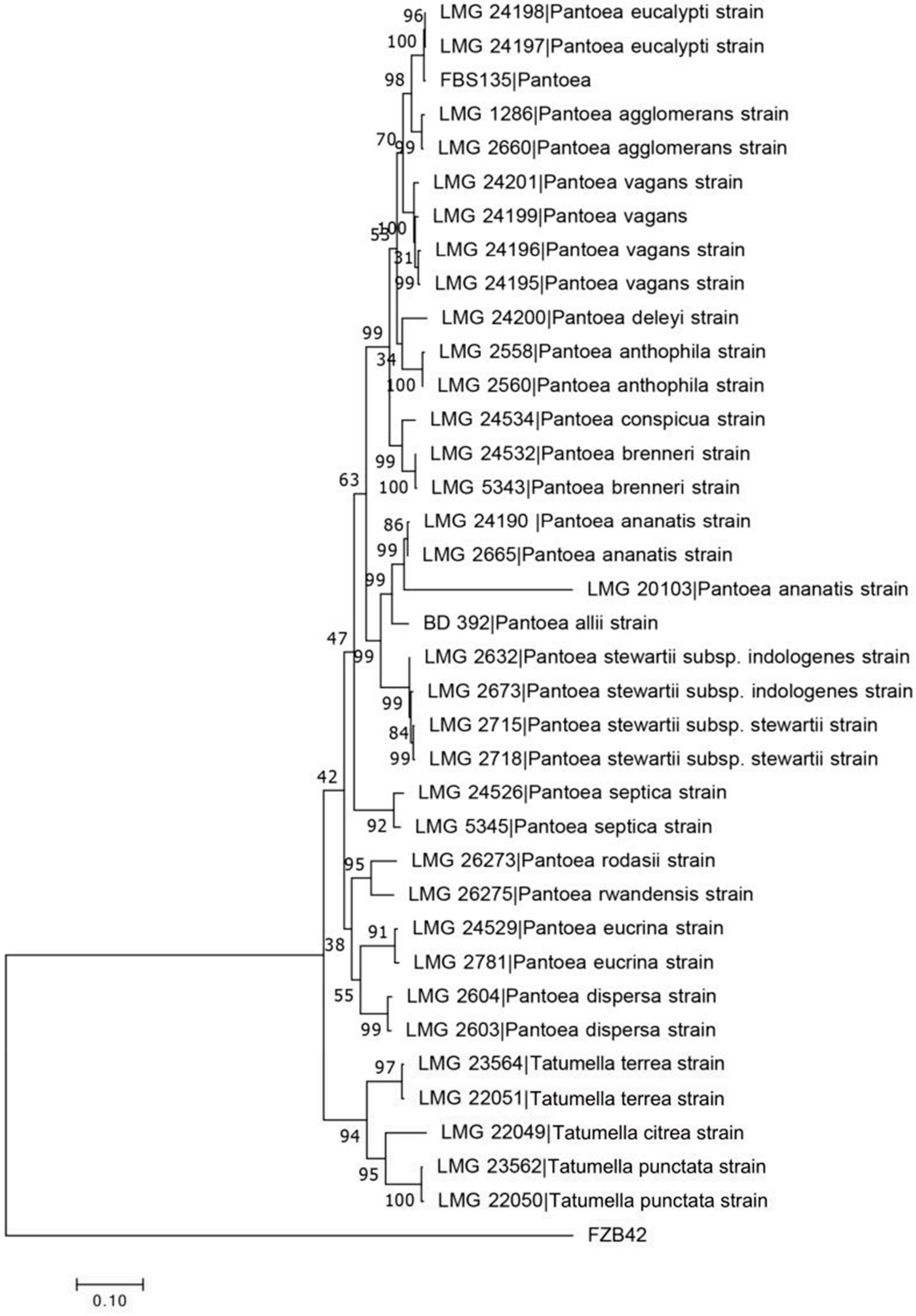

Figure S6. Phylogenetic tree constructed based on MLSA with four conserved genes (rpoB、gyrB、infB、atpD) using Maximum Likelihood method (repeated 1000 times).

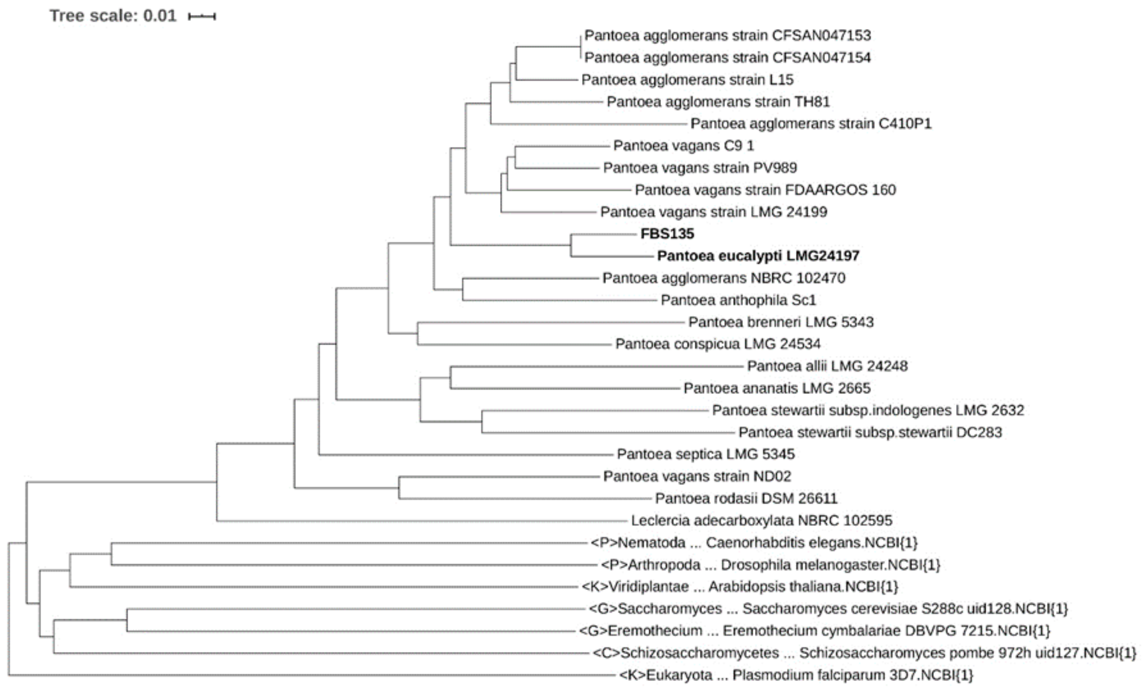

Figure S7. Phylogenetic analysis based on the whole genome sequence of FBS135
